# Multi-unit response correlations along the tonotopic gradient and within isofrequency laminae of the inferior colliculus

**DOI:** 10.1186/1471-2202-14-S1-P169

**Published:** 2013-07-08

**Authors:** Dominika Lyzwa, J Michael Herrmann

**Affiliations:** 1 Dept. of Nonlinear Dynamics, Max Planck Inst. for Dynamics and Self-Organization, Göttingen, 37077, Germany; 2 Institute of Perception, Action and Behaviour, University of Edinburgh, Edinburgh, EH 8 9AB, UK

## 

Within the central auditory system, the central nucleus of the inferior colliculus (ICC) is a station where all stimulus information converges. Furthermore, it is a target for auditory prostheses due to its tonotopic order and surgical accessibility [1]. To better understand the functional organization of this important nucleus, response similarity along the frequency gradient and within iso-frequency laminae were investigated. Furthermore, the neural discrimination was compared between pooled simultaneous and non-simultaneous responses.

Vocalizations, being natural stimuli and showing a variety of spectral and temporal modulations, are particularly interesting and can elicit responses which are not triggered by pure tones, clicks, white noise or other artificial simplified sounds [2]. Conspecific vocalizations (at intensity levels of 30-70 db) were presented monaurally to guinea pigs while recording multi-unit activity from the contralateral ICC. Data were simultaneously recorded from 32 positions either along the tonotopic gradient using double shank electrodes or within two iso-frequency lamina using double tetrode electrodes.

We investigated response similarity along these two dimensions of the ICC for multi-unit thresholded spike trains, local field potentials and voltage traces. We evaluated the correlation dependencies of multi-unit pairs on their spatial distance, difference in best-frequency and similarity of the frequency tuning curves. The presence of a distributed code would imply that the interplay of several multi-units leads to better representation of the complex sound. We have tested this by comparing neural discrimination between pooled sets of simultaneously recorded responses to non-simultaneously recorded ones. The latter was obtained by randomly shuffling the trials. We used linear discriminant analysis for the classification. The extracted features were firing rates of time windows over the duration of the longest vocalizations.

**Figure 1:**
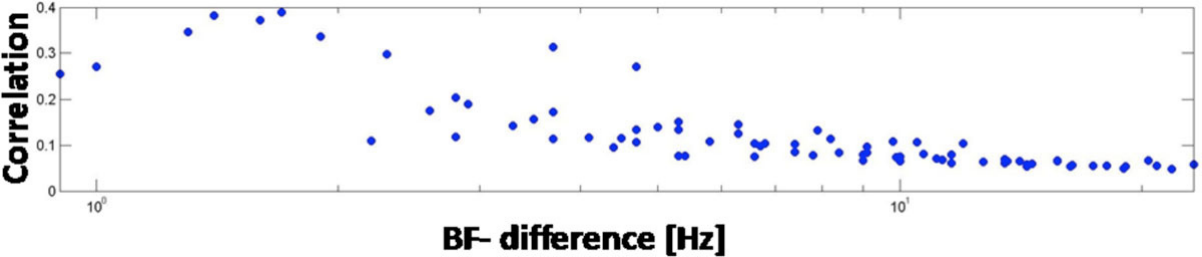
**Correlation for multi-units along the tonotopic gradient versus logarithmically scaled difference in best frequency**. Correlation decreases with BF-distance.

We found that the averaged correlation of single spike-trains is much lower than correlation of poststimulus time histograms (PSTH). This is partly due to the high trial-trial variability which is averaged out in the PSTHs. Correlation decreases with distance ( 'μm' and 'best frequency') along the tonotopic gradient, see Figure 1. Within an iso-frequency lamina though it does not decrease with μm-distance. This redundancy within the iso-frequency laminae but not along the tonotopic gradient has implications for arrays to electrically stimulate the ICC.

There is no significant difference in neural discrimination performance for simultaneous and non-simultaneous responses which could be found from the multi-unit responses.
